# Current Trends on Glomerulosclerosis Regression

**DOI:** 10.25122/jml-2020-0006

**Published:** 2020

**Authors:** Marilena Stoian, Victor Stoica

**Affiliations:** 1. Clinic of Internal Medicine, Dr.I.Cantacuzino Hospital, Bucharest, Romania; 2. “Carol Davila” University of Medicine, Bucharest, Romania

**Keywords:** Renin-angiotensin system, plasminogen activator-inhibitor-1, renal fibrosis, glomerulosclerosis, aldosterone

## Abstract

The role of the renin-angiotensin system in hypertension and end-organ damage has long been recognized. Angiotensin l converting enzyme inhibitors are superior to other antihypertensive agents in protecting the kidney against progressive deterioration, even in normotensive persons. Likewise, angiotensin II type 1 receptor antagonists improve or even reverse glomerulosclerosis in rat animal models. These findings suggest that Angiotensin II has nonhemodynamic effects in progressive renal disease. The renin-angiotensin system is now recognized to be linked to the induction of plasminogen activator-inhibitor-1, possibly via the AT4 receptor, thus promoting both thrombosis and fibrosis. Interactions of the renin-angiotensin system with aldosterone and bradykinin may impact both blood pressure and tissue injury. The beneficial effect on renal fibrosis of inhibiting the renin-angiotensin system likely reflects the central role that angiotensin has in regulating renal function and structure by its various actions. This article explores the renin-angiotensin-aldosterone system with plasminogen activator-inhibitor-1 interaction and the potential significance of these interactions in the pathogenesis of progressive renal disease and remodeling of renal sclerosis.

## Introduction

### Angiotensin and PAI-1. A Link between vasoactive and thrombotic systems

Plasminogen activator-inhibitor-1 (PAI-1) is the primary physiological inhibitor of tissue plasminogen activator (tPA), and urokinase-like plasminogen activator (uPA), both of which activate plasminogen to plasmin, thus promoting fibrinolysis and proteolysis, and also activate other matrix metalloproteinases. Angiotensin induces PAI-1 via its metabolite Ang IV which binds to the AT4 receptor in vascular smooth muscle cells and bovine aortic endothelial cells in vitro. Angiotensin induction of PAI-1 in vitro was found to be direct in the early phase, with a later component dependent on the co-induction of TGF-b by angiotensin. [1, 2]. Further, increased activity of the renin-angiotensin system (RAS), whether by exogenous infusion of physiologic amounts of Ang II or by endogenous increase linked to the ACE (angiotensin-converting enzyme) DD polymorphism increases PAI-1 levels in humans with no effect on tPA. [3]. PAI-1 activity is also genetically modulated by the common 4G/5G polymorphism located -675 base pairs from the transcription start of PAI-1. Patients homozygous for the 4G allele have increased PAI-1 levels, and also increased risk for cardiovascular disease. Compound homozygosity (i.e., ACE D/D + PAI-1 4G/4G) for ACE and PAI-1 polymorphisms that have been linked to increased cardiovascular disease and renal disease risk was associated with an increased incidence of macroangiopathic disease in diabetic patients. This may relate to the linked effects of PAI-1 and RAS to promote thrombosis and fibrosis. Indeed, inhibitors of RAS significantly reduced thrombus formation in an animal model. Increased PAI-1 has also been associated with fibrosis. PAI-1 expression was tightly correlated with sites of glomerular injury in a radiation model where thrombosis progresses to glomerulosclerosis. Decreased injury in animal models was associated with maneuvers that decreased PAI-1 by treatment with angiotensin-converting enzyme inhibitors (ACEI) or angiotensin II subtype 1 receptor antagonists (AT1RA). The modulation of PAI-1 by ACEI also occurs in humans. Inhibition of angiotensin using ACEI significantly decreased PAI-1 antigen and activity in patients following acute myocardial infarction, with no effect on tPA antigen levels. Thus, choosing a RAS inhibitor, whether the intervention affects AT4, which at least in vitro induces PAI-1, or augments bradykinin, which stimulates tPA, could potentially have a profound impact on the balance of thrombosis/fibrosis versus fibrinolysis/ extracellular matrix (ECM) degradation (see below).

### Interactions of RAS and Aldosterone

Ang-II may also affect sclerosis via aldosterone. The addition of aldosterone antagonism over angiotensin inhibition alone provided additional benefit on glomerulosclerosis in animal studies. Aldosterone antagonism alone also decreased vascular injury in the stroke-prone hypertensive rat model. Importantly, aldosterone enhanced angiotensin induction of PAI-1 in vitro. In animal studies, in the nonhypertensive radiation nephropathy model, spironolactone, an aldosterone receptor antagonist, ameliorates sclerosis. This finding was not linked to effects on blood pressure or proteinuria but was tightly associated with decreased PAI-1 expression.

These data demonstrate that inhibition of aldosterone can decrease PAI-1 in vivo, and suggest that targeting of both angiotensin and aldosterone may be necessary for optimal effect on PAI-1 and progression of glomerulosclerosis.

### Can the regression of disease-related sclerosis be achieved?

In addition to increased matrix synthesis, decreased ECM proteolysis contributes to progressive renal fibrosis. PAI-1 inhibits not only fibrinolysis but also proteolysis, by inhibiting the activation of plasminogen activators. Plasmin can cleave most ECM proteins, and both tPA and uPA play essential roles in vascular remodeling, angiogenesis, and tumor metastasis. tPA primarily affects fibrinolysis, whereas uPA has less affinity for fibrin but avidly degrades the matrix. PAI-1 expression usually is present in very low levels in the kidney and is expressed in vitro in many cells, including endothelial and visceral epithelial cells [9]. PAI-1 is increased in vascular injury settings, whether thrombotic or fibrotic. Increased PAI-1 levels, whether due to the functional 4G/4G polymorphism of the PAI-1 gene promoter or due to other causes, are associated with cardiovascular disease. TGF-b 1 effects of inducing fibrosis may also, in part, relate to PAI-1 actions: TGF-b 1 induces PAI-1 to a greater extent than uPA in cultured endothelial cells, thus promoting fibrosis. Renal biopsy studies in humans show that using ACEI not only slows the progressive loss of the glomerular filtration rate (GFR) but also prevents ongoing structural injury. In a small study of diabetic patients treated with either ACEI or beta-blockers, repeated renal biopsies were performed. Over three years, there was a slight increase in the afferent arteriolar medial matrix with a beta-blocker, while no increase was seen when using ACEI. In another study, ACEI prevented interstitial expansion in hypertensive patients with diabetic nephropathy [1, 4]. Even more dramatic results, with regression of glomerulosclerosis and interstitial fibrosis, were achieved in diabetic patients with nephropathy, whose diabetes was cured by pancreas transplantation. Repeated biopsies over a 10-year interval showed regression of mesangial expansion, more patent glomerular loops, and a proportional decrease in tubulointerstitial fibrosis [5].

Experimental models have shed light on some of the mechanisms involved in achieving the regression of glomerulosclerosis. Spontaneous resolution of mesangial matrix accumulation occurs in the anti-Thy-1 model with attendant changes in cell proliferation and increasing metalloproteinase activity. Regression of matrix expansion resulted from pancreatic transplantation in a rat model with diabetes [6, 7, 10]. Delayed onset treatment in the puromycin aminonucleoside model of glomerulosclerosis with either ACEI or low protein diet was also inferred to regress glomerulosclerosis by comparison of cohorts of animals sacrificed at different time intervals [8, 15].

Increased PAI-1 expression in sclerotic sites and decreased PAI-1 was linked to the resolution of sclerosis. Rats with regression also had an improved renal function. These findings implicate the inhibition of PAI-1 by a high dose of AT1RA or ACEI, resulting in increased matrix degradation and the regression of glomerulosclerosis. The link between PAI-1 expression and sclerosis was also demonstrated in the radiation nephropathy model, a nonhypertensive model of early endothelial injury followed by late sclerosis. PAI-1 mRNA expression by in situ hybridization was closely associated with sites of glomerular injury, assessed by serial section morphologic analysis. PAI-1 mRNA in glomeruli was localized in the injured mesangial and endothelial areas, with focal expression in glomerular visceral and parietal epithelial cells [11]. Thus, autocrine effects are involved, since these cells also express receptors for angiotensin (AT1 and possibly AT4 receptors). Treatment with AT1RA or ACEI significantly inhibited the up-regulation of PAI-1 mRNA without affecting tPA or uPA expression. Importantly, kidney sclerosis was prevented by treatment with either ACEI or AT1 RA.

### Can the regression of age-related sclerosis be achieved?

Recently, it was found that existing age-related glomerular and vascular sclerosis in rats could be remodeled, with regression and decreased collagen content induced by starting AT1RA treatment in aging rats. Aging sclerosis was accompanied by increased apoptosis in tubular and interstitial cells in the kidney, which was abolished by the AT1RA treatment [12-14]. PAI-1 and TGF-beta increase with aging, while remodeling and regression were associated with decreased PAI-1 and TGF-beta. Thus, these data support that both decreased ECM synthesis, in part due to decreased TGF-beta, and increased ECM degradation, determined by the inhibition of PAI-1, contribute to the regression of sclerosis.

## Conclusion

These data demonstrate that regression of biopsy-proven glomerulosclerosis can be achieved in various experimental settings. The potential importance of the RAS in renal fibrosis is underscored by the effectiveness of therapies that aim to inhibit its various actions, including induction of PAI-1. Ongoing studies will establish which of these recent provocative findings from animal models are relevant to human diseases and may lead to optimal therapies to forestall progression and perhaps even induce regression of sclerosis.

## Conflict of Interest

The authors declare that there is no conflict of interest.
